# Emergence of a butterfly: the life experiences of type 1 diabetes Taiwanese patients during the 16–25 years old transition period

**DOI:** 10.1080/17482631.2020.1748362

**Published:** 2020-04-15

**Authors:** Yueh-Tao Chiang, Hsing-Yi Yu, Fu-Sung Lo, Chi-Wen Chen, Tzu-Ting Huang, Chi-Wen Chang, Philip Moons

**Affiliations:** a School of Nursing, College of Medicine, Chang-Gung University, Tao-yuan, Taiwan; b Division of Pediatric Endocrinology & Genetics, Department of Pediatrics, Chang-Gung Memorial Hospital, Tao-yuan, Taiwan; c Department of Nursing, Chang-Gung Memorial Hospital, Tao-yuan, Taiwan; d Division of Pediatric Endocrinology & Genetics, Department of Pediatrics, Chang- Gung Memorial Hospital, Tao-yuan, Taiwan; e College of Medicine, Chung-Gung University, Tao-yuan, Taiwan; f School of Nursing, National Yang-Ming University, Taipei, Taiwan; g Department of Neurology (Dementia Center), Chang-Gung Memorial Hospital, Tao-yuan, Taiwan; h Department of Public Health and Primary Care, KU Leuven-University of Leuven, Leuven, Belgium; i Institute of Health and Care Sciences, University of Gothenburg, Gothenburg, Sweden; j Department of Pediatrics and Child Health, University of Cape Town, Cape Town, South Africa

**Keywords:** Type1 diabetes, life experiences, transition; adolescent; young adults; nursing; qualitative

## Abstract

**Purpose**: To explore the life experiences of patients with type 1 diabetes transition from adolescence into adulthood in Taiwan.

**Methods**: Descriptive phenomenological design was used. Fourteen participants were individually interviewed using a semi-structured interview.

**Results**: The life experiences of patients with type 1 diabetes transition from adolescence into adulthood experience a metamorphosis from awareness of responsibility to figuring out a way to care for themselves. Six themes emerged: (1) hibernation: awareness of responsibility; (2) emergence: attempts to take responsibility; (3) perseverance: encountering difficulties; (4) anxiety: multiple worries; (5) hesitation: back-and-forth,” and (6) exit: finding a way out.”

**Conclusions**: During the transition phase, the participants experienced the trials of various situations. Regardless of whether they are able to independently bear the responsibilities of self-management, they all hope to turn around the challenges of disease control and take ownership of their disease. Like a butterfly that emerges from a cocoon, they hoped to overcome the dangers of taking flight through trial and error and navigating the world. The results of this study can serve as a reference for clinical care and developing localized intervention strategies targeted to the transition period between adolescence and young adulthood.

## Introduction

Type 1 diabetes (T1D) is a disease that requires strict lifelong monitoring and blood glucose control to ensure physical and mental well-being [1]. The disease tends to develop in children and adolescents and is one of the most common chronic diseases in childhood (Cooper; Garvey et al., 2012). Worldwide, there is a trend of prevalence and incidence (International Diabetes Federation, 2016). For example, the incidence rate in individuals under 19 years old in Taiwan is 5.79/100,000 (Jiang et al., 2012). The number of T1D patients accounts for 3/100 of all diabetes patients, and approximately 10,000 patients receive treatment each year (Chen & Yang, 2014). With the increase in prevalence and incidence, it is expected that the demand for care during the T1D transition period between adolescence and early adulthood will also increase.

Transition is a process triggered by critical events and changes in individuals or environments. There are four types of transitions: developmental, situational, health-illness, and organizational (Chiang et al., 2016; Schumacher & Meleis, 1994). Developmental transitions are complex involving a predictable series of biologically determined stage of growth and psychosocial maturations, such as the drive of the secondary sex characters and the pursuit of self-identity in adolescence. Situational transitions are the changes in various educational and professional roles, such as transition from a high school student to a college student. Health–illness transitions are the processes that individuals or their families transit from health to disease or disease to health, such as the transition process of T1D patients from hospitalization to discharge due to complications. Organizational transitions represent transitions in the environment, such as the transition process of T1D patients from paediatric clinic to adult clinic, and the care model will change form family-centred to self-aided care as they grow up (Chiang et al., 2016). A subjective sense of well-being, role mastery, and wellbeing of interpersonal relationships are important indicators of successful transition (Schumacher & Meleis, 1994).

The transition from late adolescence to early adulthood is one of the most stressful periods in life. In addition to changes owing to physical and psychological development, individuals may also face complexities such as separation from home, sexual relationships, marriage, pregnancy, and employment. These issues are almost always among first major life experiences (Arnett, 2000; Santrock, 2017). T1D patients aged 16–25 years must endure both these complexities and self-care for diabetes. This often results in unimaginable pressure (2010Kansas Communities That Care Student Survey (Administration); Chang et al., 2017; Dovey-Pearce et al., 2005; Ersig et al., 2016; Rasmussen et al., 2011) and decreases their control of blood glucose (Miller et al., 2015; Ritholz et al., 2014; Rollo et al., 2014). In addition, patients may not regularly visit the outpatient clinics for follow-ups, leading to the early occurrence of comorbidities, increased numbers of disease-related hospitalizations, and accelerated deterioration from disease and impacts on quality of life (Ersig et al., 2016; International Diabetes Federation, 2016; Rollo et al., 2014). T1D patients in the transitional period need to reach a consensus with and have the support of primary caregivers and medical personnel to improve disease control outcomes (Ersig et al., 2016; International Society for Pediatric and Adolescent Diabetes, 2014). Understanding the experiences of T1D patients during the transitional period will allow for the development of interventional strategies that meet their needs to improve quality of care (Chiang et al., 2016). Therefore, studies on experiences during the transitional period of T1D patients are important for improving the effectiveness of their care.

Researches in Europe and the USA have shown that the overall experience during the T1D transitional period is a process of becoming independent, while also relying on the support of others; it is a very complicated process that requires a lot of adaptation. In addition to needing to establish a personal understanding of the disease, patients need to painstakingly integrate disease care into daily life (Abdoli et al., 2017; Karlsson et al., 2008). Failure to self-manage the disease is the biggest source of stress during the transitional period (Ersig et al., 2016; Karlsson et al., 2008). Nocturnal hypoglycaemia and long-term comorbidities cause deep anxiety and can even interfere with sleep (Ersig et al., 2016). Other issues that patients may face include deconstruction and reconstruction of interpersonal relationships, beginning sexual relationships, work stress and lack of disease control after entering the workplace, pregnancy risk, and increased economic burden caused by termination of genetic medical insurance (Chang et al., 2017; Pyatak et al., 2014; Rasmussen et al., 2011). This stage is also a time when it is easy for patients to discontinue medical treatment. Lack of comprehensive care, negative medical experiences in the adult clinic, awkwardness in surrounding disease care in the presence of peers, competing demands, loss of relatives, neglect or abuse, and substance dependence are important factors that impact the discontinuance of medical treatment for T1D patients during this stage (Garvey et al., 2014; Perry et al., 2012; Pyatak et al., 2014). Based on the above studies, T1D patients in the transitional period face overwhelming pressure, and consequently may discontinue medical treatment, or need increased support. However, these studies were conducted in Western countries, and there are significant differences in culture, geography, medical resource distribution, insurance systems, and medical care policies between Eastern and Western countries. With respect to care models, in addition to medical personnel, case managers, health educators, dietitians, and social workers participate in T1D care in Taiwan, but mainly in paediatric care models (Chiang et al., 2016). However, patients in the transitional period have different health needs than children. Moreover, care plans that meet their developmental needs have not been designed. The experiences of T1D patients during the transitional period in Taiwan likely differ from those discussed in the literature on Western contexts, and thus merit investigation. Ignoring the experiences and needs of patients can easily lead to poor interventional outcomes (Allen & Gregory, 2009; De Beaufort et al., 2009). Therefore, this study uses phenomenological research methods to understand the experiences of T1D patients during the transitional period between ages 16–25.

## Methods

### Design

Using descriptive phenomenology by applying interview techniques, the research participants are allowed to move from themselves to the object, and through the process of interacting with themselves, their experiences living with T1D can be raised to the level of consciousness as much as possible and fully described (Mu, 1996).

### Context

The study was conducted at the Division of Metabolism and Endocrinology within the Paediatric Department of a Medical Centre, one of the main referral hospital in Taiwan. The clinic serves approximately 200 T1D patients aged between 16 and 25. In Taiwan, there is lack of a national consensus guideline for how to organize healthcare of patients with T1DM during adolescence into adulthood. In our study, most of the patients do their follow-up every month. The services offered to them include medical reviews and examinations, such as HbA1C and disease management plans, diabetes education, ophthalmology (annually) and nutritionist (bimonthly) consultation.

### Participant selection

Purposive sampling was adopted for this study, with the following inclusion criteria (Garvey et al., 2012): individuals with a T1D diagnosis confirmed by an endocrinologist before the age of 16 and who have been suffering from the disease for over 6 months; (Cooper) between the age of 16 and 25 years old (International Diabetes Federation, 2016); no other metabolic diseases, chromosomal abnormalities, or major injuries (Jiang et al., 2012); can communicate in Mandarin or Taiwanese; and (Chen & Yang, 2014) adult study participants agreed to record interviews and signed letters of consent. In the case of minors, legal representative agreed to the study and signed consent letters simultaneously. The exclusion criteria were patients with autism, cognitive impairment, and speech disorders, because phenomenological studies rely on in-depth statements to reveal personal experiences related to the object of the study.

After analysing the narrative data from the thirteenth interview, it showed no new themes. To confirm data saturation, the researchers conducted another interview with the fourteenth participant. Finally, the data did show thematic saturation (Mu, 1996). Characteristics of the study participants are shown in Table I.

**Table I.** Characteristics of the study participants

Patient code	Age at time of interview (years)	Course of disease (years)	Sex	Height(cm)	Weight(kg)	BMI	Insulin administration	Two most recent HbA1 C measurements (%)	Total interview time (min)
A	19	16.5	F	164	56 (pregnant)	20.8	Pen	11–12	43
B	16	0.75	F	155	62	25.8	Pen	13–14	41
C	25	15	F	150	42	18.7	Pump	7–7.5	58
D	16	7.25	M	177	97	31	Pump	6.3–6.6	60
E	21	16	F	153	55	23.4	Pen	14–15	45
F	22	18	M	170	63	21.8	Pump	6.5–7	43
G	19	16	M	169	63	22.1	Pump	7–7.2	68
H	22	15	F	158	49	19.6	Pen	7–7.8	46
I	21	6	F	168	59	20.9	Pen	10–11	28
J	16	8	M	168	55	19.4	Pen	10–12	58
K	17	4	M	163	59	22.2	Pen	10–13	55
L	18	15	M	160	44	17.1	Pen	8.4–8.6	53
M	22	2.75	M	175	62	20.2	Pen	8.6–9.8	54
N	22	10	F	165	48	17.6	Pen	16–17	67

## Data collection

The study period was from December 2017 to June 2018. Semi-structured, in-depth interviews were used for data collection. The first author, who was the data collector, has abundant experience in T1D care, received rigorous training in qualitative research, and published several qualitative research articles in international journals. After obtaining the consent of respondents (or legal representatives for minors), interviews were conducted and recorded in the paediatric ward conference room without distractions. The process was adjusted based on the interests and wishes of the respondents, and the respondents were not guided to obtain open information. Each interview was limited to a single respondent and lasted between 28 and 67 min. Preliminary interview guidelines were prepared based on the literature review. From those, formal interview guidelines were developed, discussed among three experts, and revised and reviewed by the members of the Institutional Review Board. Further, a child psychologist and child psychiatrist were invited to review the guidelines. The final interview guidelines are shown in Table II.

**Table II.** Interview guidelines

1. How do you feel suffering from Type 1 diabetes? Are there any differences between the present and the past?
2. What changes occurred in your life after entering puberty? What are the effects of these changes on you?
3. In the face of the aforementioned changes,(1) What important relevant decisions have you made?(2) How does suffering from Type 1 diabetes affects your important decisions?
4. What kind of problems do suffering from diabetes currently cause you? What solutions have you thought of or do you use? What are the results?
5. Please discuss your interactions with your primary caregiver and medical staff?
6. What assistance for disease care do you feel is most required during the transition period from adolescence to adulthood?
7. Have you thought of transferring to adult outpatient for treatment? What are the reasons?
8. What suggestions do you have for medical staff?

### Data analysis

The descriptive phenomenological method of Giorgi was used to analyse research data (Giorgi, 1985), and the procedure and examples of the analysis are shown in Table III.

**Table III.** An exemplar of the processes of data analysis

**Step 1**: Reading the text to obtain a sense of the narrative **Step 2**: Extracting the meaning units **Step 3**: The meaning units were examined to gain a sense of the experiences and comprehensively understand them	**Step 4**: Meaning units were transformed into psychological language	**Step 5**: Specific subcategories were. related to general themes	
**Meaning unit**	**Psychological language**	**Subcategories**	**Themes**
“My classmates stigmatized me by saying that diabetes is infectious. Their parents did not want them to play with me as they were afraid that something would go wrong with me. I did not know how to explain and could only put up with the bullying and discrimination.” (N14)	Disease stigmatization resulting in bullying	3.3 Negative labels	3. Perseverance: Encountering difficulties
“During high school, sometimes my relationship with my father was somewhat affected. My father did not understand my disease, and he would be very upset when hyperglycaemia or hypoglycaemia occurred. When I was unable to go to school, he would even think that I was finding excuses. My teachers were unable to understand that I would be very upset when I had hyperglycaemia or hypoglycaemia. Sometimes I would feel absent-minded and these people did not understand. This caused my relationship with my father to be irreversibly damaged and I did not feel like going to school.” (D11)	Others are unable to understand the feelings of suffering from the disease, resulting in estrangement	3.4 Interpersonal problems	

### Rigour

The selection criteria for this study were clear. The data collectors had long-term exposure to T1D patients. During the data collection period, they conducted recorded interviews using the spirit of phenomenological reduction and remained faithful to the original attitudes of phenomenology (Mu, 1996). After the interview ended, a verbatim draft and reflective record were completed within 24 h. Analysed data were confirmed by peer verification of the content, and research data and analytical procedures were stored in their entirety for verification, thereby establishing the credibility, transferability, dependability, and confirmability of the study to maintain research rigour (Lincoln & Cuba, 1985).

### Ethical considerations

After review by the Institutional Review Board of a medical centre in Taiwan, they assigned the IRB case number 201701733B0 to this study. At the time of patient recruitment, the researchers clearly stated the purpose of the study, methods of data collection, and participants’ rights in a language they fully understood. After confirming the patients’ complete understanding and obtaining consent forms, the interviewer conducted analysis and categorization in an encoded manner. All research data were locked in a file cabinet that could only be opened by the first author.

## Results

The results of the study showed that T1D patients during the transitional period between 16 and 25 years of age experience a metamorphosis from an awareness of responsibility to finding a way out for themselves. We identified six themes: “Hibernation: Awareness of Responsibility,” “Emergence: Attempts to Take Responsibility,” “Perseverance: Encountering Difficulties,” “Anxiety: Multiple Worries,” “Hesitation: Back and Forth,” and “Exit: Finding a Way Out.” All patients looked forward to controlling the disease regardless of their current state of self-management, and they hoped to turn around the challenges of disease control and take ownership of it. Like a butterfly that emerges from a cocoon, they hoped to overcome the dangers of taking flight through trial and error and navigating the world. See Table IV for the results of the analysis.

**Table IV.** Themes and subcategories

Theme	Subcategories (number of respondents)	Description
1. Hibernation: Awareness of Responsibility	1–1 Physical changes (N = 10) 1–2 Self-awareness (N = 4) 1–3 Advice from others (N = 8)	1–1 Awareness of responsibilities due to appearance of secondary sexual characteristics 1–2 Environmental causes of self-awareness and the need to shoulder responsibility 1–3 Reminders by others and taking responsibility when growing up
	1–4 Peer comparison (N = 8)	1–4 Comparison with friends and wanting to shoulder responsibility
2. Emergence: Attempts to Take Responsibility	2–1 Opportunistic demonstration (N = 6)	2–1 Taking chances to strive to perform and obtain trust
	2–2 Recovering control (N = 6)	2–2 Recovering autonomy through hard and soft approaches
	2–3 Breaking out of shackles (N = 3)	2–3 Remaining indifferent and carrying out actions according to one’s wishes
	2–4 Practicing decision making (N = 7)	2–4 Attempting to make decisions for the disease through encouragement and support
3. Perseverance: Encountering Difficulties	3–1 Reality shock (N = 10)	3–1 Feeling helpless because reality does not equate to what is imagined 3–2 Feeling depressed due to the realization that it is easy in theory, but difficult in practice 3–3 Disease stigmatization resulting in ostracism or bullying 3–4 Experiencing interpersonal problems due to lack of understanding of the disease
	3–2 Easy in theory, but difficult in practice (N = 11)	
	3–3 Negative labels (N = 4)	
	3–4 Interpersonal problems (N = 7)	
4. Anxiety: Multiple worries	4–1 Blood glucose worsening (N = 10) 4–2 Financial difficulties (N = 8) 4–3 Short lifespan (N = 8) 4–4 Obstacles in forming a family and looking for employment (N = 7) 4–5 Inheritability of type 1 diabetes (N = 8)	4–1 Feeling anxious due to poor glycaemic control 4–2 Economic pressure due to insufficient subsidies or insurance 4–3 Fear of premature death 4–4 Fear that employment, marriage, and having children will be obstructed 4–5 Worry that type 1 diabetes will be inherited to children
5. Hesitation: Back and Forth	5–1 Shifting back and forth between independence and dependence (N = 10) 5–2 Hovering between reality and ideals(N = 7) 5–3 Entanglement between the intrinsic and extrinsic (N = 4)	5–1 Shifting back and forth between independence and dependence 5–2 Hovering between reality and ideals 5–3 Struggling between intrinsic tangible health and extrinsic image and temptation
6. Exit: Finding a Way Out	6–1 Positive attack (N = 5) 6–2 Dare to try (N = 4) 6–3 Precautions against the unknown (N = 7) 6–4 Thought adjustment (N = 6) 6–5 Making requests (N = 5) 6–6 Entrusting spirituality (N = 5) 6–7 Setting goals (N = 4)	6–1 Directly stating disease to avoid questioning or embarrassment 6–2 Ignoring risks and adjusting routine care or treatment according to one’s wishes 6–3 Proposing strategies to reduce damage caused by disease 6–4 Change of thinking to accept facts and decrease frustration 6–5 Acknowledge shortcomings and needs and proposing ideas or seeking help 6–6 Obtaining spiritual peace through beliefs or religious support 6–7 Formulating disease management goals and slowly taking responsibility

### Hibernation: awareness of responsibility

Patients who transition from adolescence to early adulthood experience sexual changes, increased time spent with classmates, continuous reminders from parents and medical staff, or family environment changes. These events caused patients who were primarily cared for by parents to become aware of their own responsibilities. The changes are akin to a caterpillar transforming into a butterfly inside a cocoon after undergoing metamorphosis. Although it appears static, it has started to change, prepares to leave behind its parents’ care, and starts a new phase of life.

Many transition period patients became aware that they have grown up due to physiological changes that prompt them to act like adults and shoulder the responsibility of taking care of themselves.

My voice has broken, and I should act like a man. Being sick is my own affair. Therefore, I should shoulder my own responsibility and control glycated hemoglobin.”(D100)

Patient A, who was 19 years old, suffered from diabetes for 3 years and had poor glycaemic control. However, due to pregnancy, she actively requested to be hospitalized to adjust her insulin dose and started to change her behaviour and attitude.

When I was pregnant and decided to give birth to my child, I knew that I was going to be a mother and should not let poor glycemic control cause my child to be deformed. I can no longer be willful and must act like an adult to protect my child. (A67)

Some patients were forced to become independent due to family circumstances. Therefore, when they were sick, they knew that they had no other way out and must shoulder their own responsibilities.

My mother had undergone dialysis before I developed diabetes, and my father is old. Therefore, when the doctor said that I had diabetes, I knew that only I could take care of myself. (B65)

Reminders from parents, physicians, nursing staff, health education teachers, and schoolteachers—or comparison with classmates, friends, or siblings—provided a basis for patients to become aware of his/her responsibilities or a desire to mature. Becoming an adult meant assuming responsibility for his/her disease care during the transition period.

### Emergence: attempts to take responsibility

During the transition period, patients no longer followed orders or instructions, but instead made use of daily opportunities to care for themselves. Through decision-making, they recovered control of their care. They began to ignore the exhortation of caregivers and achieved disease care through their own methods or practised decision-making under the encouragement of their parents. Both active attack and passive acceptance were starting points for patients to attempt to shoulder disease care responsibilities, which are akin to a butterfly that slowly spreads its wings after breaking out of the cocoon.

Some transition period patients considered being alone as an opportunity to demonstrate their capabilities and maintain glycaemic control within the ideal range to earn the trust of parents or caregivers.

When I have the opportunity, I will take the chance to let people believe in me. An example is at school or going out with my classmates, I will measure blood glucose and inject myself on schedule and obtain a nice score at home. After a few times, parents will naturally believe that you are capable. (J109)

Although most parents recognized that their children had grown up, they were still worried and reluctant to let their children be responsible for complex daily care for diabetes or disease-related decisions. This caused patients who were eager for responsibility to employ both hard and soft approaches to transfer decision-making from caregivers.

Once, I threw a large tantrum as I did not wish to tell my teachers and classmates. My parents were frightened, but I only wished to inform them that I have grown up, and I should be able to decide for myself. They should no longer treat me as a child and let me take care of myself. (F74)

Some patients adopted an indifferent attitude and ignored the caregiver’s exhortation due to either a communication failure or desire not to quarrel. To demonstrate their autonomy, these patients ate based on their preferences, and only measured blood glucose when they were unwell.

No matter what other people say, I am responsible for my own problem. This is my body. For many years, I can feel myself and I test my blood glucose when I am unwell.(K68)

Some caregivers slowly started to transfer care responsibilities during this period, so that patients could assume responsibilities with the support and encouragement of caregivers and others, and practice decision-making for disease-related care.

They had many options for me to choose and would ask me for my opinion. With their encouragement, I would attempt to make decisions. Although sometimes I would worry whether my decision was right or wrong, I would attempt to decide. (C26)

### Perseverance: encountering difficulties

During the process of leaving the primary caregiver and shouldering routine disease care, patients inevitably encountered some accidents or setbacks. Through these events, they understood that some people will be clueless and realized that knowledge and actions are not as easy as imagined. In addition, they did not have sufficient capabilities to clarify misunderstandings or stigma towards people living with diabetes. They developed misunderstandings and conflicts with others due to the disease. These experiences caused patients to suffer, which is akin to flying butterflies that encounter their natural predators or various obstacles in flight.

Patients were usually unable to predict the occurrence of possible interfering events. During moments of device malfunction, loss of medicines, or accidents that disrupted care routines, patients usually experienced shock and difficulties. Although these were sporadic events, some patients began to doubt themselves, which fostered a lack of confidence.

Once, the pump motor was broken, and I was unable to inject the medicine, causing my blood glucose level to increase, which was uncontrollable. This was a feeling of frustration. During the period when the pump was not working, I had to directly inject myself, and I forgot how to do it as I do not do it often. I felt helpless because of this. The pump can also malfunction, and this fact is cruel. It caused me to crumple. I really overestimated myself. (G34)

The patient thought that he/she was extremely detailed in life and that maintaining blood glucose stability was not difficult. After these challenges, the patient realized that there were too many external temptations, and it was difficult to overcome his/her own desires without the care or supervision of others. The patient understood that diabetes care was easy in theory, but difficult in practice.

I thought that I was very clear on what I could eat and what I could not eat. Only when I ate outside did I realize that it was not easy. I would want to eat the same foods as my classmates and could not control my desire to drink beverages. In addition, I did not have time to monitor blood glucose due to classes, causing my blood glucose to spike. This caused me to be extremely depressed. I also did not wish to be like this and felt that it was difficult to manage. I can only say that knowing is one thing, but doing is a more difficult matter. (I45)

During this period, the patient was extremely mindful of weird looks from others and fearful of negative labels. His/her classmates misunderstood the disease and believed that diabetes was infectious. Moreover, the patient’s parents worried that accidents might occur and their child would not be able to shoulder the responsibility. Classmates’ parents asked their children keep a distance from the patient, causing the patient to feel ostracized or bullied.

My classmates stigmatized me by saying that diabetes is infectious. Their parents did not want them to play with me, as they were afraid that something would go wrong with me. I did not know how to explain and could only put up with the bullying and discrimination. (N14)

The patient often encountered interpersonal difficulties due to T1D with family members, friends, classmates, teachers, colleagues, and medical staff. Patient D’s father and teachers believed that he used the disease as an excuse, and this resulted in estrangement with his father and dropping out of school.

During high school, sometimes my relationship with my father was somewhat affected. My father did not understand my disease, and he would be very upset when hyperglycemia or hypoglycemia occurred. When I was unable to go to school, he would even think that I was making up excuses. My teachers were unable to understand that I would be very upset when I had hyperglycemia or hypoglycemia. Sometimes I would feel absent-minded, and these people did not understand. This caused my relationship with my father to worsen, and I did not feel like going to school. (D11)

### Anxiety: multiple worries

Although many patients were still under the care of their caregivers as they became older, they started to become more involved in self-care, have increased self-concern, or have misconceptions of T1D. They developed disease-related worries, such as worsening blood glucose levels, financial difficulties, obstacles in forming a family and seeking employment, disease transmission to their children, and premature death.

Normally, patients personally experienced disease complications before they worried about the consequences of poor disease control. Hypoglycaemia and hyperglycaemia were patients biggest worries, and most patients only thought about long-term complications occasionally.

I inject myself with insulin at 9 pm. Every time I finish injecting myself, I will be fearful that I will not wake up once I sleep if I develop hypoglycemia at night and will be sent by an ambulance to the emergency department. This causes me to lose sleep. (B52)

Patients in the transition period began worrying about whether they could shoulder future financial burdens caused by the disease. Patients who used insulin pumps worried that they would be unable to pay the cost of consumables every month, which is around 330 USD. Patients who developed the disease at an early age were unable to purchase private insurance and worried about whether the national health insurance was sufficient to support disease expenses.

I developed the disease in elementary school and am unable to buy other insurance except for the national health insurance. In the future, I worry that I will not be able to shoulder these costs if I develop eye or kidney complications, and I do not know if the national health insurance will still be around and how much it can help me. (D13)

Disease often causes people to think about death. Although 16–25-year-old patients are young, most had thought about dying. This is because they fear sudden or premature death due to poor glycaemic control. Some patients who experienced hypoglycaemia often were also unable to control their thoughts, and thought about death and the fear of the unknown.

I have thought about what happens after I die. I fear that I will suddenly die if my blood glucose is too high or too low, particularly so as I often suffer from hypoglycemia. I also do not know, and I fear that I will suddenly die. When I am in a daze, images of death will flash in my brain, causing me to be frightened. (B54)

Obstacles to seeking employment, making friends with the opposite sex, and marriage were worries felt by the patients. In addition, more than half of the patients, regardless of their gender, worried about diabetes inheritance, which affected their intention to get married and have children. Some adult patients even decided not to have children out of regard for their children’s health.

### Hesitation: back-and-forth

During the process of learning about health autonomy and disease management, patients found that independence was not as positive as they imagined, or the difference between reality and ideals and intrinsic-extrinsic entanglement caused them to shift back and forth between independence and dependence, hover between reality and ideals, and entangle between the intrinsic and extrinsic.

After experiencing independence, patients felt that it was good to be cared for and longed to remain children. Although they had the ability to care for themselves, they selectively relied on the caregiver and continuously shifted between independence and dependence.

I could care for myself and hoped that my mother could let go. However, sometimes, I would feel shameless, particularly when the pump malfunctioned, and I asked my mom to help me inject insulin. I knew how to inject myself but did not feel like doing it. Therefore, I would ask my mother to help me inject insulin. (G35)

People always have ideals and expectations. However, patients were unable to balance ideals and reality due to actual health conditions and other factors, and they hovered between reality and ideals. Patient N described an imbalance between his/her ideals and physical condition, which resulted in fatigue.

I cannot find a balance between ideals and my real-life physical condition, and I am still searching and even tried my best to cope. I thought that nurses will treat me the best because I am a civil servant. However, there are three sessions per week, and I find that I am unable to cope as my colleagues do not understand me. I am truly exhausted. (N60)

During this stage, most patients understood the importance of disease control. However, to maintain their physique, satisfy their food desires, or maintain their relationship with their peers, they continuously struggled between intrinsic tangible health and extrinsic-specific ideas and temptations. Patient C mentioned that he/she was concerned about weight gain during puberty because food must be taken after an insulin injection. Therefore, the patient did not perform the injections and kept telling himself/herself that this would be the last time.

During my rebellious phase, I did not wish to do this on the one hand, as I knew that it affects glycemic control. On the other hand, I could not stand my weight gain. Every time, I would not inject myself so as to maintain my body weight. This was because I did not feel hungry if I did not inject myself and would not need to eat. Following that, I would keep telling myself that this would be the last time that I was indulging myself. In fact, I continuously struggled between my inner thoughts and outside temptations. (C56)

### Exit: finding a way out

The path to independence is a huge challenge filled with setbacks. Therefore, patients attempted to positively attack, dared to try, took precautions, adjusted their thoughts, made requests, entrusted their spirituality, and set goals to regulate stress and overcome problems. Regardless of whether the strategies employed were successful, they hoped that they would ultimately reverse the disadvantage of being controlled by the disease and become an expert in controlling the disease to obtain maximum physical and mental comfort.

Measuring blood glucose, performing injections, controlling diet, and using body pumps sometimes attracted glances or attention from others. Patients believed that rather than concealing or lying, they should naturally perform whatever task was required, or talk about the task as a form of positive attack to avoid unnecessary embarrassment or questioning.

You should proactively mention it and not wait for people to ask you to explain why you cannot eat carbohydrates. There is no need to lie to hide the fact that you are sick.(L35)

Some patients believed that they understood themselves better than physicians. To identify the best method of controlling their disease, they adjusted care routines based on their feelings while risking possible impact or danger due to unstable blood glucose. Some female patients did not inject themselves with insulin to control their physique and manipulated blood glucose levels to avoid scolding or nagging. Although some did not follow medical instructions for injections and attempted to alter test results, confusing results stumped medical staff, who attempted to alter insulin dosage.

For a period of time, my glycated hemoglobin increased to more than 13. This was because I felt that I understand my body best, and therefore I used my own method to attempt to not inject myself for one meal as I do not want to attract sideways glances in school. However, during breakfast and dinner I would compensate for the missed lunch dose. In the subsequent 2 years, I had found that this was untenable as my HBA1c almost reached 14. Hence, I complied with instructions and injected myself. (L16)

In senior high school, I was a bit rebellious. At that time, I did not know if I had an endocrine disorder or other problems as I started to become obese. Even if I ate very little, my body weight would keep going up. Following that, I thought about the onset of my disease, which was when I started to become very thin within one week. I thought of using that method, which was an idiotic way of starting not to inject myself. However, I saw that my blood glucose records were good. Therefore, I would select conducive timing to first measure blood glucose and not when it should be measured so that I could fake the results. The measurement records generated looked good on paper, and I would not be nagged or scolded by my physician or mother. However, the glycated hemoglobin values would tell the truth, and the results were obviously poor. (C34)

As their disease-related knowledge or interpersonal experience increased, patients were able to exercise precautions in potentially dangerous situations. An example included preparing Super Supau sports drinks during exercise as a precaution against hypoglycaemia. Some patients would also avoid situations where they would be hurt in order to prevent interpersonal harm.

You do not require every classmate or friend to care for you and will be able to know who can help you and who will harm you. Therefore, you do not need to interact with people who will hurt you because of your disease. If you do not let them know about your disease, you will not be hurt. Therefore, very few people know of my disease, except for my teachers, as I do not tell them. (E29)

When faced with the unchangeable fact that they suffered from diabetes or when they were feeling down, many patients employed different methods to change their mindset to alleviate setbacks, avoid psychological burden, or accept the fact that they suffer from diabetes.

Everybody will be ill at one point or another. Therefore, I felt that I just happen to suffer from diabetes. There are 1.7 million diabetics in Taiwan, including Type 2 diabetes. Therefore, I do not feel so lonely. (M35)

Patients were slowly able to acknowledge their shortcomings and needs and proposed ideas or sought help. They hoped to learn how to assume responsibilities with support and improve their disease management capabilities. They wanted their parents to learn to let go, so that they had an opportunity to grow up. Although most patients continued follow-up treatment in the paediatrics department due to trust in physician–patient relationships and a familiar medical environment, four patients recognized that the needs of adults are different from children and wanted assistance in identifying suitable physicians for adult care. More than half of the patients mentioned limitations with existing mobile applications (APPs) and recommended development of APPs that conform to patients’ needs during the transition period. In addition, patients mentioned the following needs: awareness development and guidance on disease knowledge, personalized and feasible diet substitution methods, parent–child communication techniques, joint consultation and treatment, disease identification cards, friendly public spaces, knowledge of T1D, suitable social subsidies, premarital and genetic counselling, psychological counselling, and employment counselling.

They should help refer us to physicians for adults after we have grown up. There is still a difference. (K63)

Medicine should be technicalized. Currently, APPs are very popular. I previously looked at two APPs for diabetes, but they are not targeted to us but are general knowledge or specialize in the elderly or type 2 diabetics. You should seek the opinions of more young people to develop something to record life activities, such as what I have done today and what my estimated blood glucose is, record where I seek treatment and who is my physician. In addition, you can share the experience of trying new foods and how much insulin is roughly needed so that people can use it as a reference. All these things can be integrated together, which will be of practical use. (G82)

When faced with difficult problems or unchangeable facts, patients used their religious beliefs to overcome suffering due to disease and obtain spiritual support and comfort.

I believe in Jesus Christ, and I will pray when I encounter difficulties to wish for hope and look for the correct direction to go forward. Praying to Christ to give me health, and I pray for my disease to be controlled. (K56)

When I feel upset due to my disease, I will tell myself that although God has closed this door, he will open another window, as a form of consolation. (H12)

To slowly improve their confidence in glycaemic control and build hope that they are able to assume the responsibilities of disease self-management, patients set specific, feasible goals, and indicated a desire to be the chief executive officer (CEO) of their disease.

After I have awakened from poor glycemic control, I told myself that I must set goals and slowly become the CEO of diabetes. I will control it, so that it no longer threatens me. (D67)

## Discussion

This study indicates that the life experiences of T1D patients from adolescence to early adulthood are like emergence of a butterfly. Participant had an awareness of responsibility of their diseases and most of them can find a way out after experiencing setbacks and anxieties, eventually they all hope to turn around the challenges of disease control and take ownership of their disease. Like a butterfly that emerges from a cocoon, they hoped to overcome the dangers of taking flight through trial and error, and navigating the world.

Regardless of developmental transition or organizational transition, most T1D patients are delineated by age, and usually occurs at late adolescence or early adulthood, around 17–19 years old (Chang et al., 2017; Dovey-Pearce et al., 2005; Ersig et al., 2016; Hilliard et al., 2014; Rasmussen et al., 2011). If age is used alone as a basis for delineation, whether the motivation for change is present or absent in the patient may be overlooked. A lack of motivation, goals, or preparations will cause patients to be unable to carry out proper health self-management (Chang et al., 2017; Chiang et al., 2016; Garvey et al., 2014). Therefore, in addition to distinguishing by age, the motivation and preparedness of patients should be assessed as factors for successful transition. Our study found that patients are aware of their own responsibilities and attempt to shoulder them, which is the period when self-care motivation and proactiveness is highest. This may be an opportunity that primary caregivers or medical staff can discuss the transfer of disease care responsibilities with patients. The earlier appearance of the secondary sex characters, more opportunities for peer comparison, and participants with higher self-awareness have a tendency to detect disease management responsibility earlier.

As adolescents learn how to become independent, we found one of the setbacks they encounter is disease stigmatization by peers and labelling. An example is the incorrect idea that diabetes is contagious. Patients in such scenarios often do not know how to explain or clarify the disease. This result is similar to the findings of Abdoli, Hardy (Abdoli et al., 2017) and Fedor, Schumacher (Fedor et al., 2017). As the community has low literacy on T1D, stigma attached to disease characteristics and conception may affect patients psychologically impact their beliefs about diabetes. In addition, transition period patients are at an inter-dependent relationship stage, regardless of whether they are entering senior high school from junior high school, entering the university from senior high school, or entering the workplace (Karlsson et al., 2008; Rasmussen et al., 2011). Therefore, understanding, encouragement, and support play an important role in autonomous behaviour, and adolescents’ self-management of T1D during the transition period (Karlsson et al., 2008). We recommend that peer- or community-level awareness activities be used to educate the aetiology of T1D and its acute management. This will help reduce the general public’s stigma towards the disease, while simultaneously promoting community and patient empowerment. In addition, assisting transition period patients with establishing interpersonal support and coping techniques with uniformed individuals is also important in patients’ ability to redefine themselves and achieve self-identity (Chiang et al., 2016).

The adolescence-early adulthood transition stage is the worst period for blood glucose control in type 1 diabetes (Miller et al., 2015; Wood et al., 2013). In our study, among the 10 participants treated with insulin pen, 90% had HbAlc values in the last two times exceed the recommended range of 7.5 gm/dl, and even 70% of them had values exceeding 10 gm/dl, which not only interfered with their sleep but also made some of them lose confidence in disease self-management due to depression. This is consistent with other research findings (Ersig et al., 2016; Fedor et al., 2017). Analysis of the causes of poor blood glucose control showed that the hormonal changes of four participants in adolescence may have increased the insulin demand, which is an important cause of blood glucose instability (Chiang et al., 2016; International Society for Pediatric and Adolescent Diabetes ISPAD Clinical Practice Consensus Guidelines 2018, 2018). Therefore, we should help adolescents understand the impact of hormonal changes on blood glucose control, in order to reduce their frustration (Chang et al., 2017; Chiang et al., 2016). For the participants in early adulthood, the main causes of poor blood glucose control are insufficient self-efficacy and more competitive needs, including inability to resist external temptation, desire to integrate with peers, a busy student life or work that interferes with the self-care of the disease, etc. Therefore, the key for helping young adult achieves the goal of self-management is to separately understand the difficulties of self-care in real life and further discuss coping strategies, so as to increase confidence over self-management of type 1 diabetes.

Previous papers have mostly showed positive coping strategies for stress adjustment during the transition period (Chang et al., 2017; Rasmussen et al., 2011), which either did not mention negative coping strategies or found that these are inappropriate or even unsafe techniques. The results of this study found that many patients can adopt positive attacks, take precautions against the unknown, adjust thoughts, make requests, set goals, and engage in other positive methods to handle stress. However, some patients are overly focused on their appearance, want to avoid glances from their peers, or believe that they understand their body better than their physicians, which causes them to ignore their prescribed medical treatments and possible consequences. These patients also adopt risky strategies and perform care routines on a whim, based on their experiences and feelings. When their glycaemic control fails to meet expectations, they manipulate blood glucose readings to obtain trust from their parents and physicians, this puts their health at risk. The existing transition period care system lacks psychological counselling and stress coping guidance (Chang et al., 2017; Garvey et al., 2014). Therefore, future assessment of disease coping and glycaemic control should pay attention to the application of negative strategies. In addition, stress management intervention should also be a goal of transition period care.

For some, religious beliefs provide support and comfort, increasing their ability to overcome disease suffering. It is just one of the methods for self-adaptation in the transition period that was discovered in this study. The study of Abdoli, Hardy (Abdoli et al., 2017) also obtained similar results: that transition period T1D adolescents believed diabetes was God’s will. This belief enabled patients to accept their disease. In addition to using spirituality to accept their disease realities, our study also found that patients wish to obtain hope from religious beliefs so that disease control can improve or that they can even recover from it. The important role of religion in coping with the treatment of chronic disease has been confirmed in many studies (Pilger et al., 2016). Therefore, spiritual support should be included in transition period care.

With regard to transition period care needs, many patients mentioned that learning how to get caregivers to let go is an important factor in reducing conflict and enabling independence. Studies in other countries have also showed that although parents know that they should transfer responsibilities, they still intervene and interfere to a considerable extent, worsening parent–child relationships (Allen et al., 2011; Dashiff et al., 2011; Ersig et al., 2016). Therefore, an in-depth examination of primary caregivers’ care experience is needed to develop intervention measures that assist them in effectively letting go and transferring disease management responsibilities to patients. In addition, the guidelines of care for the patient with T1D transition from adolescence to young adult has not yet been developed, and the paediatric care model is still adopted in Taiwan (Chang et al., 2017; Chiang et al., 2016). Most participants in this study expressed unwillingness to transition to adult care systems, because of the trusted doctor–patient relationship and the familiar environment and care mode. However, nearly 30% of the interviewees still hoped to transfer to an adult clinic, because they felt uneasy waiting with children in the paediatrics clinic. In the future, should pay attention to the issue about how to help willing patients successfully transition to adult clinic or provide an integrated care plan under the existing care mode. Lastly, patients suggested that care methods should be provided as society progresses, such as the development of APPs. They mentioned that existing APPs mostly provide general diabetes knowledge, and may not be suitable for adolescent or young adult patients, nor do they consider personal needs. Therefore, development of a specific APP that meets transitional needs is an urgently needed for T1D care. We suggest that transition period patients should be included for the design, development, and testing stages of such APPs.

## Conclusions

The results of this study showed that transition period experience of T1D patients is a metamorphosis process from hibernation to emergence, which is akin to the process of transforming from a pupa to a butterfly. During the pupa stage, patients realize patients are no longer children and use various methods to encourage their maturation. Although patients experienced setbacks, worries and hovered between independence and dependence, intrinsic and extrinsic, and reality and ideals, most were able to find an exit and self-regulate. Regardless of whether patients were currently managing their health independently, all patients hoped to successfully control their illness. One even pledged to become the CEO of his/her disease.

## Implications

The findings provide a scientific knowledge base for nursing professionals to understand the natural life experiences of patients with T1D transition from adolescence into adulthood. Our study points to several areas where healthcare for transition period patients with T1D could be improved: (1) Attention should be paid to the situation where the patients used negative strategies to face stress. (2) Counselling should be provided to assist patients in adolescence and early adulthood to establish supportive interpersonal relationships. (3) Consideration of spirituality may be included in future care plans. (4) Should pay attention to help willing patients successfully transition to adult clinic. (5) Development of a specific APP that meets transitional needs is needed for T1D care.
